# Model-informed precision dosing of vancomycin in children 3 months to 18 years of age using Australia-wide data

**DOI:** 10.1128/aac.01840-25

**Published:** 2026-05-12

**Authors:** Haiping Xu, Wenyu Yang, Tony Lai, Brendan McMullan, Daniel Yeoh, Amanda Wilkins, Zoy Goff, Xiao Zhu, Amanda Gwee

**Affiliations:** 1 Department of Clinical Pharmacy and Pharmacy Administration, School of Pharmaceutical Sciences, Fudan University 12478, Shanghai, China; 2 Pharmacy Department, The Children's Hospital at Westmead 8538, Sydney, New South Wales, Australia; 3 The University of Sydney Infectious Diseases Institute (Sydney ID) 442308 , Sydney, New South Wales, Australia; 4 Sydney Pharmacy School, Faculty of Medicine and Health, The University of Sydney 4334, Sydney, New South Wales, Australia; 5 Department of Infectious Diseases, Sydney Children’s Hospital, Randwick, New South Wales, Australia; 6 School of Clinical Medicine, The University of New South Wales 7800, Sydney, New South Wales, Australia; 7 Department of Infectious Diseases, Perth Children's Hospital 60081, Perth, Western Australia, Australia; 8 Wesfarmers Centre for Vaccines and Infectious Diseases, Telethon Kids Institute, University of Western Australia 2720, Perth, Western Australia, Australia; 9 Department of General Medicine, Royal Children’s Hospital 553270, Melbourne, Victoria, Australia; 10 Department of Paediatrics, University of Melbourne 2281, Parkville, Victoria, Australia; 11 Antimicrobials Group, Murdoch Children’s Research Institute, Parkville, Victoria, Australia; 12 Pharmacy Department, Perth Children’s Hospital, Perth, Western Australia, Australia; 13 National Key Laboratory of Advanced Drug Formulations for Overcoming Delivery Barriers, Shanghai, China; University Children's Hospital Münster, Münster, Germany

**Keywords:** external validation, vancomycin, model-informed precision dosing, pediatrics, population pharmacokinetics

## Abstract

Vancomycin is used to treat serious gram-positive infections in children; however, effective dosing information for those aged 3 months to 18 years is limited. We aimed to determine an optimized dosing strategy for this age group. A population pharmacokinetic model was developed using data from The Royal Children’s Hospital (RCH), Melbourne. Children aged 3 months to 18 years who received vancomycin were included. The model was externally validated with data from the Children’s Hospital at Westmead (CHW), Sydney Children’s Hospital (SCH), and Perth Children’s Hospital (PCH). Simulations were performed to evaluate the standard dosing regimen and propose an optimized strategy, targeting an AUC_24–48_ of 400–650 mg·h/L. An empirical loading dose was evaluated against the same therapeutic range for AUC_0–24_ and AUC_24–48_. The RCH data set included 3,687 vancomycin concentrations from 627 children. A two-compartment model with renal maturation best described the data. External validation (PCH 175, SCH 58, and CHW 170 concentrations) showed adequate predictive performance. The optimized dosing strategy achieved a target attainment of 73% versus 44% with the standard 15 mg/kg of body weight administered every 6 h. Improvement was most notable in patients <4 years, where the probability of target attainment (PTA) increased from 10.5%–48% to 66.1%–80% with the optimized regimen. A 25 mg/kg loading dose significantly increased the initial 24-h PTA to 57%, compared to 23% without a loading dose. We developed an optimized vancomycin dosing strategy that achieved the therapeutic target in 73% of children. This regimen requires prospective clinical validation.

## INTRODUCTION

Vancomycin, a glycopeptide antibiotic, is empirically used for the treatment of serious gram-positive infections in children, particularly those due to methicillin-resistant *Staphylococcus aureus* infections and coagulase-negative staphylococci (CoNS) (1). The ratio of the area under the concentration-time curve to the minimum inhibitory concentration ratio (AUC/MIC) greater than 400 is recognized as the pharmacodynamic (PD) index for efficacy (2, 3). However, the risk of acute kidney injury increases at higher exposures, particularly when the AUC exceeds 650 mg·h/L (4, 5). Given its narrow therapeutic range and risk of toxicity (nephrotoxicity and ototoxicity) with high drug exposure, therapeutic drug monitoring (TDM) is essential to ensure safe and effective treatment.

The pharmacokinetics (PK) of vancomycin in children are highly variable during childhood, influenced by renal function, weight, and postmenstrual age (PMA). Therefore, these patient characteristics must be considered in vancomycin dosing regimens (6, 7). While several high-quality studies have addressed vancomycin dosing in young infants <3 months of age (8, 9), evidence for the 3 months to 18 years population remains less comprehensive. Current vancomycin pediatric models often rely on data sets with uneven age distributions within the 3-month to 18-year range. Specifically, older children (≥12 years) represented only 11% of the cohort in the study by Kloprogge et al. (10), while Shen et al. (11) focused on a population with a median age of 2.22 years. Even a comprehensive pooled analysis by Colin et al. (12) had encountered notable data gaps in the 1–3 year age range. Furthermore, most vancomycin population pharmacokinetic (PopPK) models in the latter age group were validated using data from the same institution (11, 13), limiting their generalizability to other settings. Additionally, no large-scale population PK model has been established specifically for Australian children. Despite these modeling efforts, weight-based dosing regimens of 60 mg/kg day remain standard of care in most guidelines (14, 15); however, this dose often results in sub-therapeutic concentrations (16, 17) and fails to account for known patient-specific factors such as age and renal function.

Therefore, this study aims to develop a vancomycin PopPK model for patients aged 3 months to 18 years, validate the model using external data from three independent hospitals, and determine initial empirical vancomycin dosing regimens for children.

## MATERIALS AND METHODS

### Study population and data collection

For the development of the PopPK model, data were retrospectively collected from children admitted to The Royal Children’s Hospital (RCH), Melbourne, between March 2020 and May 2023 who were aged 3 months to 18 years, received intravenous vancomycin, and had at least one vancomycin concentration determined. Patients receiving extracorporeal membrane oxygenation therapy (ECMO), peritoneal dialysis, or renal replacement therapy (RRT) were excluded. Data were extracted from the hospital’s electronic medical records and included the vancomycin dosing regimen, demographic information, admission to the intensive care unit (ICU), and laboratory results, including serum creatinine (SCR), (Urea), and albumin (Alb). Patients lacking the aforementioned data were excluded directly, with no subsequent data imputation conducted. For external validation of the final model, data sets were obtained from three other Australian tertiary pediatric hospitals: The Children’s Hospital at Westmead, Sydney (CHW), Perth Children’s Hospital (PCH), and Sydney Children’s Hospital (SCH). TDM practices, vancomycin and serum creatinine assays, and data collection methods across institutions are outlined in Table S1.

### Population pharmacokinetic modeling

A PopPK model was developed with NONMEM (version 7.5.0, Icon Development Solutions, Ellicott City, MD, USA) using Perl-speaks-NONMEM (PsN, version 5.2.6; Uppsala University, Uppsala, Sweden) and the Pirana Modeling Workbench (version 3.0; Pirana Software & Consulting BV, USA). The first-order conditional estimation with the interaction method was used to estimate pharmacokinetic parameters and their variability. R (version 4.4.2; https://www.r-project.org/) was used for graphical outputs.

Model development is presented in Table S2. Changes in the Akaike Information Criterion, the precision of parameter estimates (relative standard errors [RSE]), and diagnostic plots were used to evaluate and compare structural one- and two-compartment models. An exponential model was applied to describe interindividual variability (IIV), while proportional, additive, and combined error models were evaluated to characterize residual variability. Inter-occasion variability (IOV) was assessed in CL to account for changes in drug clearance across multiple treatment episodes.

This study applied a standard clearance scaling model (equation 1), consistent with the approach recommended by Germovsek et al. (18) and used in our prior studies (19). This model incorporates allometric scaling of body weight (equation 2) and a sigmoidal maturation function (equation 3). Specifically, clearance and volume parameters were scaled allometrically to a 70 kg adult reference, employing fixed exponents of 0.75 and 1.0, respectively, for body weight (20). The sigmoidal maturation function, designed to quantify the age-dependent maturation of renal clearance, includes two key parameters: PMA_50_ and the Hill coefficient. PMA_50_ represents the PMA at which clearance achieves half of its maximal value, while the Hill coefficient defines the steepness of this nonlinear relationship (12, 21). An updated version of the renal maturation function, in which the total maturation F_mat_ was defined as the product of a postnatal age (PNA) transition factor (F_mat,PNA_ days) (equation 4) alongside the PMA-based maturation (weeks) (equation 3), was also evaluated based on recent physiological modeling (22, 23).

As vancomycin is renally cleared, SCR incorporated as a covariate on CL was tested *a priori* as per Colin et al. (12) (equation 5). Moreover, as our model spanned a large pediatric age range of 3 months to 18 years, SCR was standardized as age-adjusted SCR (SCR_std_) in line with the work of Ceriotti et al. (24) shown in equation 6.


(1) $$CL=TVCL\times F_{size}\times F_{Mat}\times F_{Renal},$$

(2) $$F_{size}={\left(\frac{WT}{70}\right)}^{0.75},$$

(3) $$F_{Mat}=\frac{PMA^{Hill}}{PMA^{Hill}+PMA_{50}^{Hill}},$$

(4) $$F_{mat,PNA}=1-PNA_{max}+PNA_{max}\times \left(1-e^{-\frac{\ln(2)\times PNA}{PNAT50}}\right)$$

(5) $$F_{Renal}=e^{-\theta_{SCR}\times (SCR-SCR_{std})}$$

(6) $${SCR}_{std}=-2.37330-12.91367\times ln\left(age\right)+23.93581\times {\left(age\right)}^{0.5}.$$

The impact of covariates (ICU admission, sex, Urea, Alb, and central line) on PK parameters was evaluated. An initial exploratory analysis was conducted to screen for correlations, using analysis of variance for categorical variables and linear regression for continuous covariates, with a univariate significance level of *P* < 0.05. Subsequently, significant and clinically plausible covariates were advanced to a stepwise covariate modeling (SCM) procedure, using standard criteria for forward inclusion (a decrease in the objective function value [OFV] > 3.84, *P* < 0.05) and backward elimination (ΔOFV > 10.83, *P* < 0.001) to determine the final model.

### Model evaluation

The final model was internally validated through goodness-of-fit diagnostic (GoF) plots, including individual predictions as a function of the dependent variable (DV) and conditional weighted residuals (CWRES) as a function of time and DV. Accuracy of the final model was determined using prediction-corrected visual predictive checks (pcVPC) (1,000 simulations) and bootstrap resampling (300 replicates). For the latter, median and 95% CI values were compared with original estimates to evaluate model robustness.

### External validation

The final model was applied to describe data from the validation data sets. Bayesian posterior estimates of individual concentrations were generated in NONMEM (MAXEVAL=0) using fixed population pharmacokinetic parameters. Visual inspections of GoF plots and pcVPC plots were used to validate the final model.

### Monte Carlo simulations for dosing optimization

Monte Carlo simulations were performed using the final PopPK model to evaluate the probability of target attainment (PTA) of achieving an AUC at 24–48 h (AUC_24–48_) of 400–650 mg·h/L (3, 5). The resulting median trough concentrations were also calculated as a secondary reference. A virtual pediatric cohort (*n* = 1,000 per subgroup) was generated based on age, weight, and serum creatinine values from our multi-center patient database. The SCR categories in the dosing regimens were established according to the RCH Laboratory’s standard reference ranges and further informed by the SCR distribution from a covariate database of 703 children (aged 1–18 years) at The Royal Children’s Hospital Melbourne.

For each subgroup, the following regimens were simulated as 1-h infusions every 6 h: (i) standard dosing (AUC_24–48_/MIC): a fixed dose of 15 mg/kg of body weight (RCH dosing regimen); (ii) optimized dosing (AUC_24–48_/MIC): a dose-escalation simulation was performed to identify the optimal empirical dose for each subgroup. Doses achieving a PTA of at least 95% of the maximal value were considered candidates, and the lowest among these was selected to balance efficacy and safety; (iii) loading dose (AUC_0–24_/MIC and AUC_24–48_/MIC): a 25 mg/kg of body weight loading dose was simulated in combination with the optimized dosing regimen. We assumed an MIC of 1 mg/L because the majority of *S. aureus* isolates have an MIC ≤ 1 mg/L, as reported by the European Committee on Antimicrobial Susceptibility Testing (25).

## RESULTS

### Study population

The final RCH data set used for model development comprised 3,687 plasma vancomycin concentrations from 627 patients (median 11.0 mg/L per patient: range 5–48) who had a median age of 6.8 years (range: 0.2–18.4) and a median SCR of 31.0 μmol/L (range: 10–328). A total of 27.8% of patients were admitted to ICU, and these patients had 523 (14.2%) observations. The median number of vancomycin concentrations per child was 3 (range: 1–57). External validation was performed using three data sets: PCH (175 concentrations from 35 children), SCH (58 concentrations from 17 children), and CHW (170 concentrations from 170 children). Patient demographic and clinical characteristics are described in Table 1. The distribution of sampling times relative to previous dose for both intermittent and continuous infusions is illustrated in Fig. S1.

**TABLE 1:** Patient characteristics and data distribution for the development and validation cohorts^*a*^

Number (%) or median [min, max]	Model building cohort	External validation cohort		
	RCH	PCH	SCH	CHW
Number of patients	626	35	17	170
Male, *n* (%)	344 (55.0)	26 (74.3)	12 (70.6)	92 (54.1)
Admitted to ICU	174 (27.8)	1 (2.9)	0 (0)	0 (0)
Age (years)	6.80 [0.20, 18.4]	6.30 [0.30, 18.1]	2.90 [0.30, 16.6]	1.85 [0.20, 17.1]
3 months–2 years	128 (20.4)	3 (8.6)	7 (41.2)	90 (52.9)
2–6 years	161 (25.7)	14 (40.0)	3 (17.6)	33 (19.4)
6–12years	160 (25.6)	10 (28.6)	2 (11.8)	33 (19.4)
12–18 years	177 (28.3)	8 (22.9)	5 (29.4)	14 (8.2)
Postmenstrual age (weeks)	397 [39.4, 998]	369 [52.0, 984]	191 [55.6, 906]	135 [53.0, 933]
Weight (kg)	22.9 [2.70, 115]	22.8 [3.00, 75.1]	16.8 [3.90, 127]	11.4 [2.20, 97.1]
Serum creatinine (μmol/L)	31.0 [10.0, 328]	28.0 [9.00, 82.0]	32.0 [11.0, 65.0]	24.0 [6.50, 132]
Albumin (g/L)	31.0 [12.0, 49.0]	N/A	N/A	N/A
Urea (mmol/L)	3.80 [0.350, 17.7]	N/A	N/A	N/A
Number of observations	3,687	175	58	170
Number of observations per patient	3.00 [1.00, 57.0]	5.00 [2.00, 11.0]	2.00 [1.00, 9.00]	1.00 [1.00, 1.00]
Concentration (mg/L)	11.00 [5.00, 48.0]	10.0 [3.00, 46.0]	12.0 [4.00, 43.0]	10.7 [5.20, 23.5]
Sampling time after dosing (h)	5.25 [0, 65.7]	4.20 [0, 11.0]	0.93 [0, 7.83]	5.83 [3.18, 8.88]

^*a*^ ICU, intensive care unit; RCH, Royal Children’s Hospital; N/A, not available; PCH, Perth Children’s Hospital; SCH, Sydney Children’s Hospital; and CHW, The Children’s Hospital at Westmead.

### Population pharmacokinetic modeling and internal model evaluation

**Fig 1:**
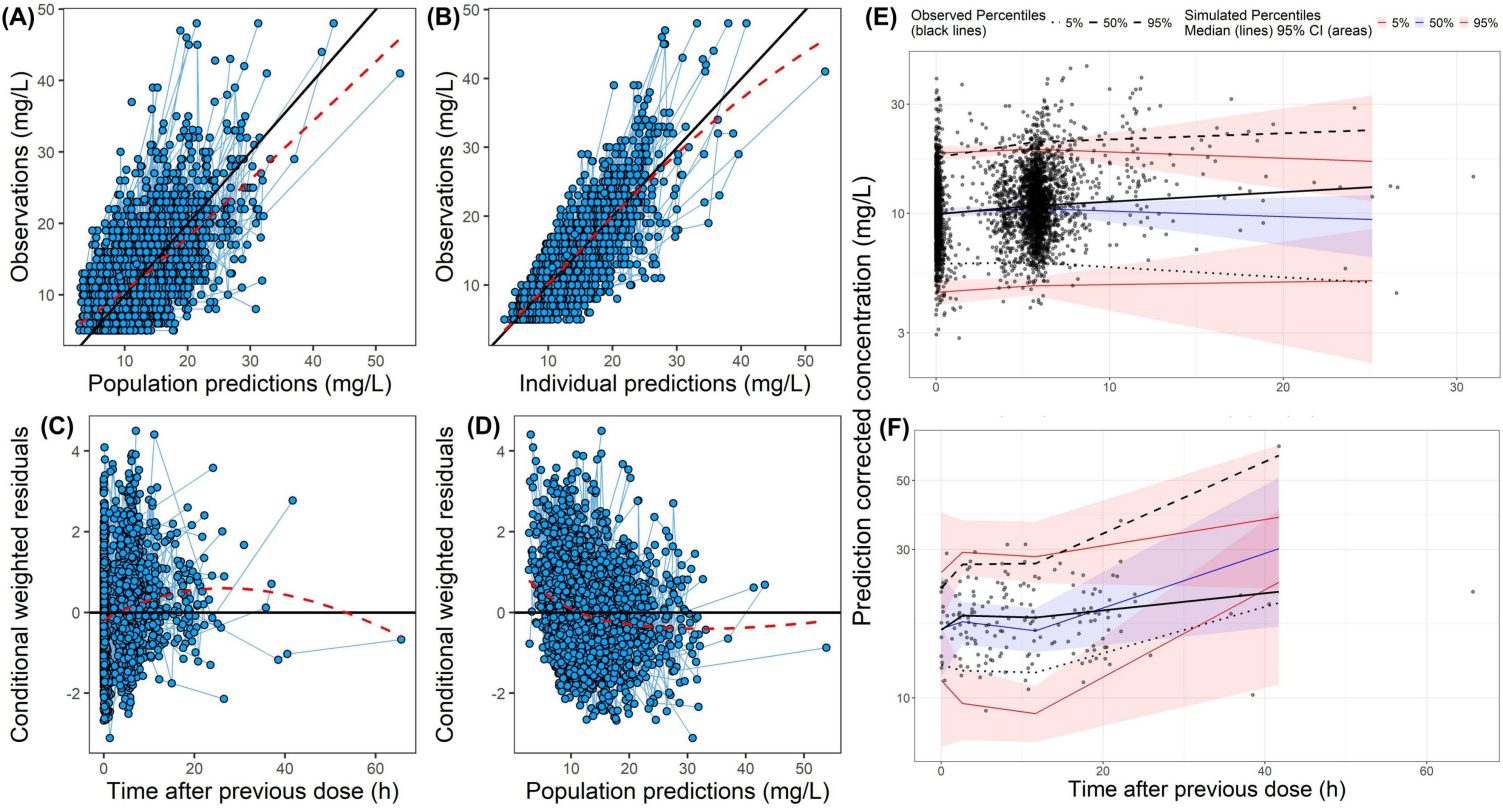
Goodness-of-fit plots and prediction-corrected visual predictive check plots of the final model for vancomycin. (**A–D**) Goodness-of-fit plots: (**A**) population predictions (PRED) vs observations; (**B**) individual predictions vs observations; (**C**) conditional weighted residuals vs time after previous dose (h); and (**D**) CWRES vs PRED. For panel **A**, the black solid line is the reference line for *Y* = *X,* and the red dashed line is the loess curve of these data. “Time after previous dose” is defined as the time interval between the start of the most recent drug administration and the collection of the blood sample. (**E** and **F**) Prediction-corrected visual predictive check plots for intermittent infusion (**E**) and continuous infusion (**F**). The black line represents the median observed data, and the two black dotted and dashed lines represent the 5^th^ and 95^th^ percentages of observed data, respectively. The light blue and light red areas represent the 95% CI for the median and the 5^th^ and 95^th^ percentiles of the simulated data, respectively.

**Fig 2:**
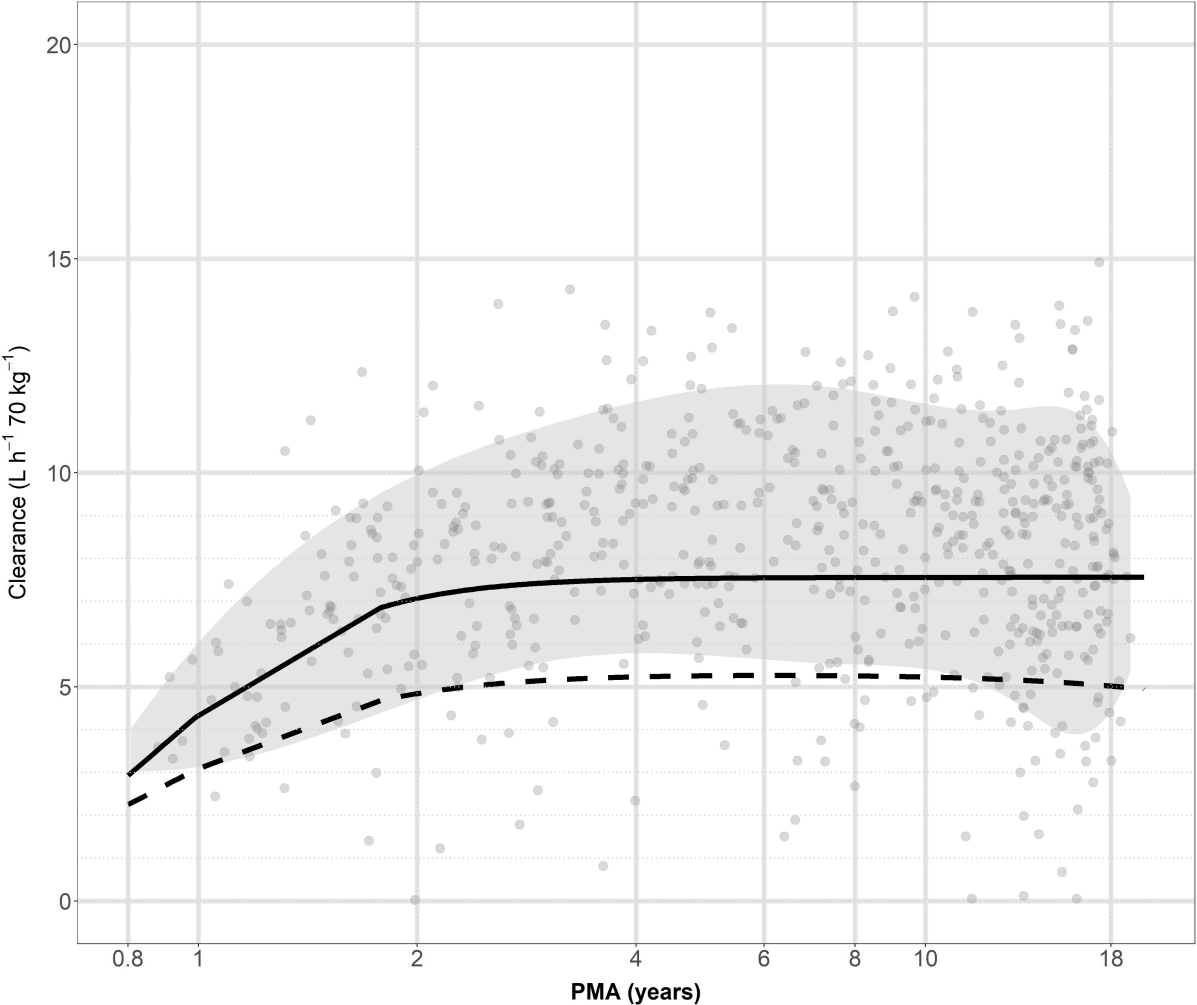
Standardized clearance (CL_std_) (L h^−1^ 70 kg^−1^) for vancomycin throughout pediatric life. The solid black line represents the typical population clearance predicted by the final model. Individual *post hoc* standardized clearance (CL_std_) estimates are depicted as solid gray circles. The gray shaded band indicates the interval between the 10^th^ and 90^th^ percentiles of the data. The dashed black line serves as a reference, illustrating the vancomycin maturation function reported by Colin et al. (12). PMA, postmenstrual age.

A two-compartment model with first-order elimination best described the data (Fig. 1). As shown in Table 2, all parameters were precisely estimated (RSE < 40%) and closely matched the bootstrap median values, indicating that the final model was robust. Allometric scaling of body weight (CL, Q: 0.75; V1, V2: 1.0) significantly improved model fit (ΔOFV = −895.74). As the Hill coefficient and PMA_50_ in the maturation equation could not be accurately estimated, they were fixed according to Rhodin et al. (12, 26), achieving significant model improvement (ΔOFV = −34.57). As shown in Fig. 2, the typical clearance values standardized for PMA (L/h/70 kg) are presented for the entire patient cohort. The model demonstrated that clearance reached 90% of its maximum value by 2 years PMA and subsequently plateaued after 3 years PMA. Furthermore, incorporating a nonlinear function that relates SCR_std_ to age on CL resulted in a more robust model fit (OFV = −1,517.11). Additionally, the inclusion of IOV on CL improved the model’s fit (ΔOFV = −296.47). After SCM, there were no significant covariates to be included in the model.

**TABLE 2:** Final pharmacokinetic parameter estimates^*a*^^,^^*f*^

Fixed effect parameters	Estimated value (RSE)	Bootstrap median [2.5^th^–97.5^th^ percentile]
CL^*b*^ (L/h/70 kg)	7.56 (2.8%)	7.56 (7.13–7.95)
Effect of creatinine on CL	0.0188 (3.9%)	0.0188 (0.0163–0.0212)
Hill	3.4 FIX	N/A
TM_50_ (weeks)	47.7 FIX	N/A
*V*1^*c*^ (L/70 kg)	85.6 (0.9%)	85.2 (79.3–92.2)
*Q*^*d*^ (L/h/70 kg)	1.30 (14.7%)	1.30 (0.94–1.68)
*V*2^*e*^ (L/70 kg)	286 (31.2%)	273 (157–483)
Inter-individual variability (CV%) (RSE) [Shrinkage]		
CL	22.1% (8.5%) [31%]	21.9% (18.0%–25.8%)
*V*1	32.9% (11.8%) [51%]	32.2% (23.8%–40.1%)
Inter-occasion variability (CV%) (RSE) [Shrinkage]		
CL	18.7% (8.2%) [41%]	18.5% (15.5%–21.5%)
Residual unexplained variability (RSE) [Shrinkage]		
Proportional error (%)	23.3% (2.3%) [9%]	23.2% (22.3%–24.3%)

^*a*^ CL, clearance; *V*1, volume of distribution of central compartment; *Q*, intercompartmental clearance; *V*2, volume of distribution of peripheral compartment; TM50, the time to reach 50% of adult clearance, the value was fixed from Rhodin et al. (26); Hill, defining the steepness of the nonlinear relationship, the value was fixed from Rhodin et al. (26); RSE, relative standard error; N/A, not available.

^*b*^ $CL=TVCL\times {\left(\frac{weight}{70}\right)}^{0.75}\times \frac{PMA^{3.4}}{PMA^{3.4}+47.7^{3.4}}\times F_{SCR}$.

^*c*^ $V1=TVV1\times \left(\frac{weight}{70}\right)$.

^*d*^ $Q=TVQ\times {\left(\frac{weight}{70}\right)}^{0.75}$.

^*e*^ $V2=TVV2\times \left(\frac{weight}{70}\right)$.

^*f*^ $F_{SCR}=e^{\left(-\theta_{SCR}\times \left(SCR-\left(2.37330-12.91367\times \ln(age)+23.93581\times (age{)}^{0.5}\right)\right)\right)}$.

### External validation

**Fig 3:**
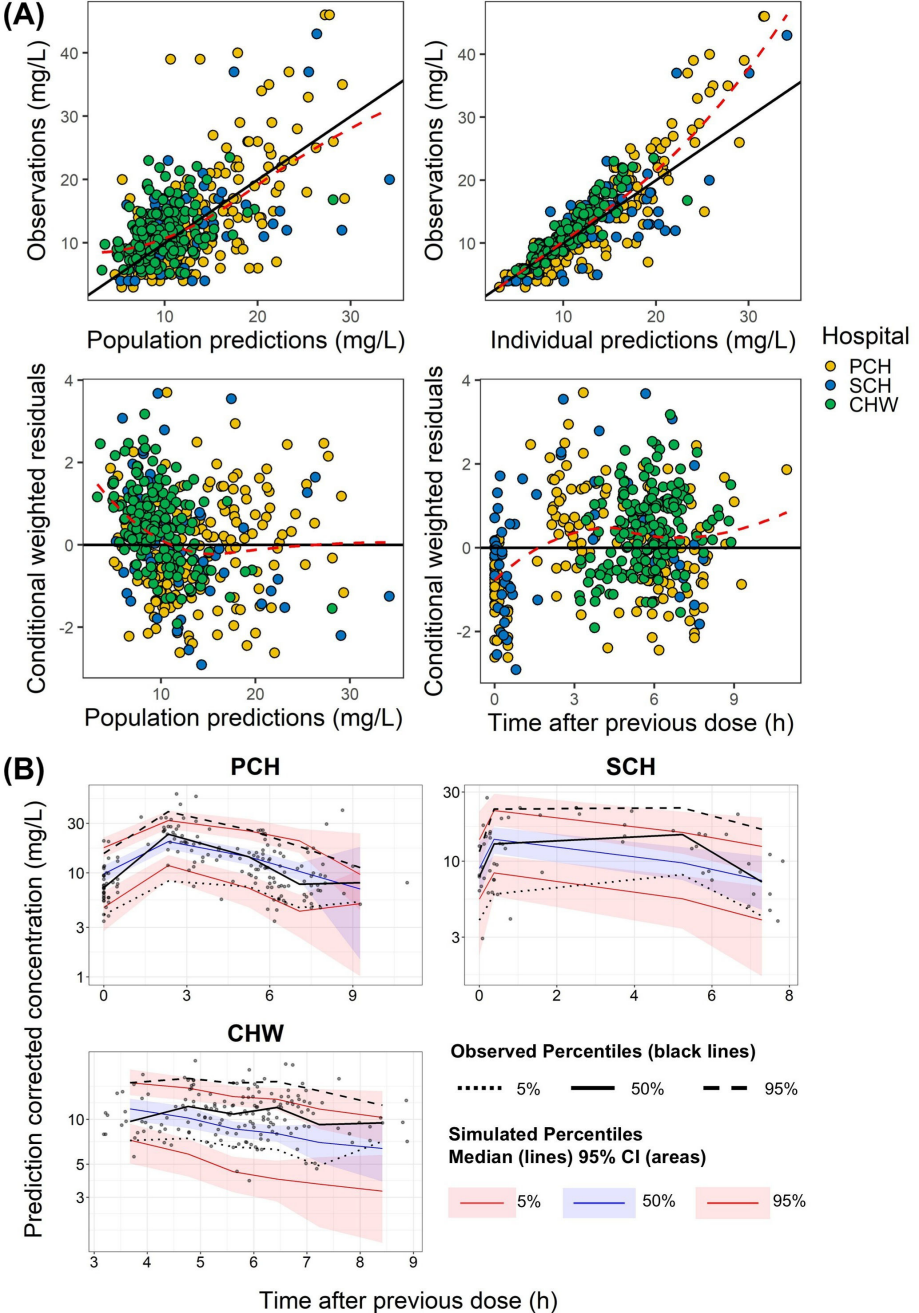
Goodness-of-fit plots and prediction-corrected visual predictive check plots of external validation for vancomycin. (**A**) Goodness-of-fit plots, including four panels: population predictions (PRED) vs observations; individual predictions vs observations; conditional weighted residuals vs time after previous dose (h); and CWRES vs PRED. For **A**, the black solid line is the reference line for *Y* = *X,* and the red dashed line is the loess curve of these data. “Time after previous dose” is defined as the time interval between the start of the most recent drug administration and the collection of the blood sample. (**B**) Prediction-corrected visual predictive check plots of Perth Children’s Hospital (PCH), Sydney Children’s Hospital (SCH), and Children’s Hospital at Westmead Sydney (CHW) data sets. The black line represents the median observed data, and the two black dotted and dashed lines represent the 5^th^ and 95^th^ percentages of observed data, respectively. The light blue and light red areas represent the 95% CI for the median and the 5^th^ and 95^th^ percentiles of the simulated data, respectively.

External validation demonstrated that the final model had adequate predictive performance, as shown in the GoF and pcVPC plots (Fig. 3). Across all three validation sets, the pcVPC analyses indicated the final model had satisfactory predictive ability.

### Monte Carlo simulations

Table 3 presents the detailed results and PTA for the standard and optimized dosing regimens across different age and SCR subgroups. The optimized regimen demonstrated a substantial improvement, with an overall PTA of 73%, compared to 43.6% for the standard regimen. This higher PTA (65.0%–80.4%) was consistently observed across all subgroups. The median (range) dose for younger children (3 months–10 years) was 84 mg/kg of body weight/day (64–88), maximum 830 mg/day. For adolescents (10–18 years), the daily dose was lower at 56 mg/kg/day (40–76), maximum, 1,165 mg/day. Notably, the median trough levels achieved with the optimized regimen remained within the commonly accepted range of 5–20 mg/L for all subgroups (27).

**TABLE 3:** Simulated dosing regimen for different age groups^*a*^^*,^c^*^

Age	Serum creatinine (μmol/L)	Standard dosing, q6h		Optimized dosing, q6h	
		Regimen (mg/kg of body weight) (PTA^*b*^, %)	Trough levels (mg/L), median (IQR)	Regimen (mg/kg of body weight) (PTA^*b*^, %)	Trough levels (mg/L), median (IQR)
3 months–1 year	10–30	15 (10.5)	7.92 (5.34–10.84)	22 (66.1)	11.86 (8.67–15.45)
1–4 years	10–30	15 (10.8)	7.82 (5.51–10.43)	22 (65.9)	11.72 (8.30–15.01)
	30–50	15 (47.6)	12.07 (9.07–15.69)	19 (79.9)	15.14 (11.59–19.60)
4–10 years	20–40	15 (28.0)	9.58 (6.84–13.17)	21 (76.6)	13.79 (9.93–17.76)
	40–60	15 (66.5)	14.01 (10.53–18.21)	16 (80.4)	15.56 (12.20–19.60)
10–16 years	30–60	15 (46.6)	11.96 (8.36–15.82)	19 (71.9)	14.54 (10.92–18.91)
	60–80	15 (72.3)	18.49 (14.06–23.72)	13 (79.2)	16.31 (12.56–19.65)
16–18 years	50–80	15 (63.4)	14.41 (10.71–19.59)	15 (70.6)	14.82 (11.32–19.26)
	80–110	15 (47.1)	23.27 (18.16–29.51)	10 (66.6)	15.85 (12.53–19.54)

^*a*^ q6h, dosing every 6 h; PTA, probability of target attainment.

^*b*^ PTA for AUC_24–48_.

^*c*^ For each renal function sub-group (based on serum creatinine ranges), 1,000 virtual subjects were simulated.

**Fig 4:**
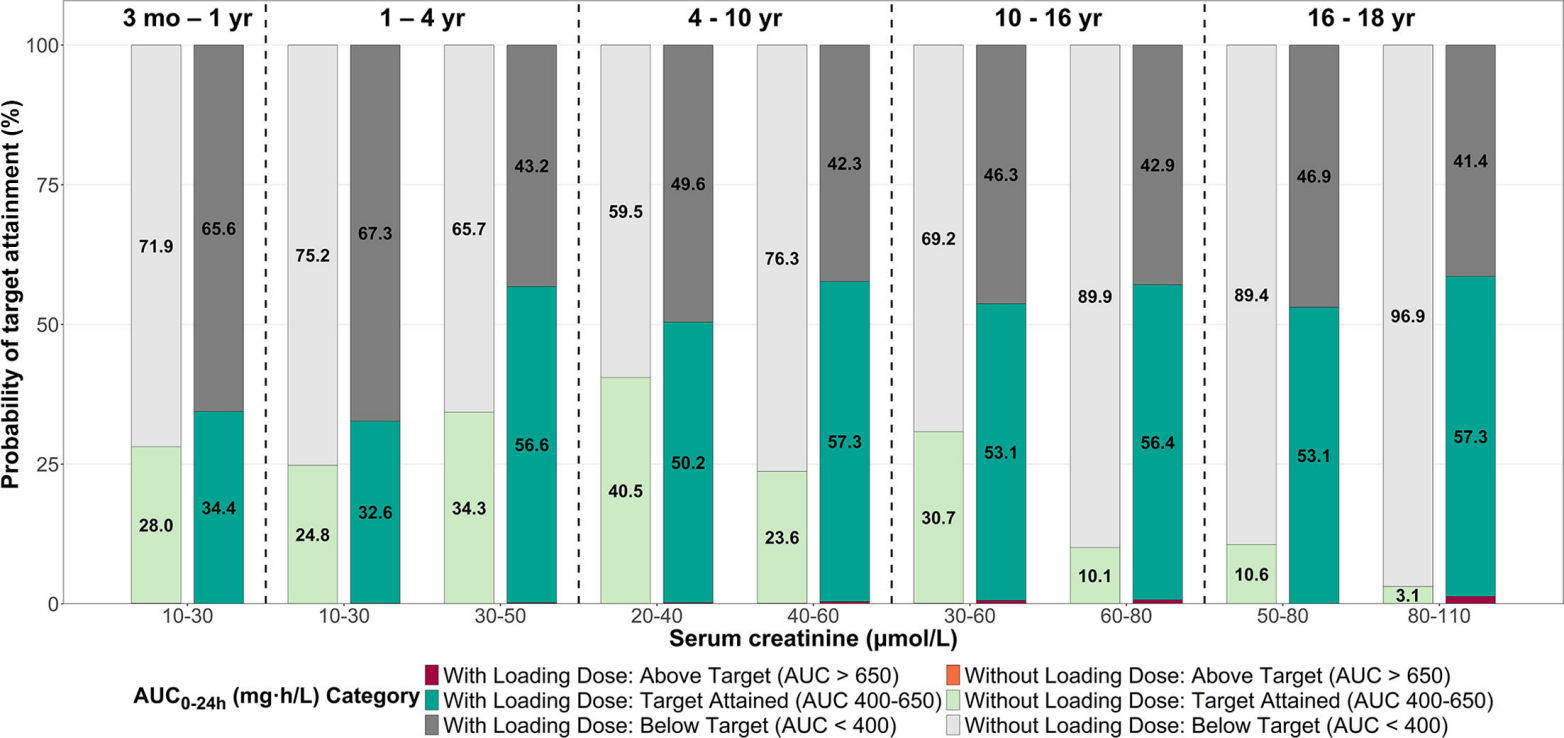
Distribution of initial AUC (AUC_0–24_) categories by age and serum creatinine The bars represent the PTA for AUC_0–24_ with and without a 25 mg/kg of body weight loading dose. Vertical dashed lines and standardized age labels (mo, months; yr, years) are used to distinguish pediatric age categories. For each renal function sub-group (based on serum creatinine ranges), 1,000 virtual subjects were simulated.

Figure 4 illustrates PTA for different subgroups at a loading dose of 25 mg/kg of body weight, targeting an AUC_0–24_ of 400–650 mg·h/L. Comparison of dosing regimens with and without a loading dose revealed that the loading dose increased PTA across all subgroups, ranging from 32.6% to 57.3% with a loading dose versus 3.1% to 40.5% without. Notably, the proportion of patients with supratherapeutic exposures (AUC_0–24_ > 650 mg·h/L) was 3.5% with the loading dose, which was comparable to the 0.3% observed in the group without a loading dose. Beyond the initial 24 h, simulations for the subsequent 24–48 h period demonstrated that a loading dose of 25 mg/kg of body weight followed by the proposed regimen showed stable PTA (60%–82%) across all age and weight subgroups (Table S3). The risk of overexposure remained below 11% in all subgroups.

## DISCUSSION

In this study, we developed a robust PopPK model for vancomycin from a large Australian pediatric cohort. The model’s robustness was confirmed through external validation using data from three independent centers. Our proposed optimized dosing regimen (Table 3) achieved a high PTA compared with the standard 60 mg/kg of body weight/day dosing regimen (73% vs 43.6%). Notably, within the same age group, the required dose varied by nearly 14.5%, underscoring the importance of considering SCR in vancomycin dosing strategies.

Our study highlights the significant limitations of a “one-size-fits-all” approach, using a 60 mg/kg/day vancomycin dose for all children, consistent with previous studies. In a study of 49 pediatric cancer patients, a dose of 60 mg/kg of body weight/day achieved a target AUC_24_/MIC ≥ 400 in only 21.5% of the virtual subjects (28). Our age- and creatinine-stratified dosing regimen recommends higher weight-based doses for younger children than for adolescents. This finding is consistent with the results of Shen et al. (29), who reported that adolescents had a higher PTA for an AUC_0–24_/MIC ≥ 400 than young children (79.5% vs 57%). It also directly reflects the established maturational trajectory of vancomycin clearance, which peaks in early childhood and subsequently declines toward adult levels during adolescence. Such a physiological change is supported by the CL-PMA relationship identified in both our model (Fig. 2) and by Colin et al. (12), whose model throughout life also showed that 90% of adult clearance is reached by 2 years PMA. This trend is also well in line with GFR maturation by O’Hanlon et al. (23). Our simulations support a median dose of 84 mg/kg of body weight/day in children aged 3 months to 10 years and 56 mg/kg of body weight/day in those over 10 years. This is similar to a large study (*n* = 702) by Le et al. (30), who proposed an age-stratified regimen: 70 mg/kg of body weight/day (<12 years) and 60 mg/kg of body weight/day (≥12 years), which achieved an AUC/MIC ≥ 400 target in approximately 75% of children.

A 25 mg/kg of body weight loading dose effectively doubled the initial PTA without excessive exposure, as fewer than 5% of participants had an AUC_0–24_ > 650 mg·h/L and less than 11% exceeded this threshold for AUC_24–48_. This is consistent with a retrospective study showing that children who received a median loading dose of 24.4 mg/kg of body weight were more likely to achieve the target initial trough than those who did not (37.0% vs 10.4%) (31). The target trough range in that study was 10–15 and 15–20 mg/L for non-invasive and invasive infections, respectively. Although studies in adults have also found that a loading dose improves the attainment of the therapeutic target without significantly increasing the risk of nephrotoxicity (32), there are no clinical data to demonstrate that this leads to improved clinical outcomes. The Neovanc trial identified higher rates of abnormal hearing screening without clear clinical benefit when an optimized vancomycin regimen, including a 25 mg/kg of body weight loading dose, was used in infants aged ≤ 90 days (33). The authors suggested that this finding may have been influenced by other factors, including more frequent dosing (12-hourly) in infants with post-menstrual age < 29 weeks and the small sample size. Nevertheless, pending longer-term outcome data, these results suggest that loading doses should be used with caution in infants younger than 90 days. These safety concerns have not been consistently found in young infants in other studies (34–37). Furthermore, there is a paucity of studies evaluating the association between vancomycin loading doses and hearing impairment in older children aged 3 months to 18 years. Large, prospective, long-term studies that consider several vancomycin treatment factors (including vancomycin cumulative dose, loading dose, duration, and exposure) are needed to determine if there is a true association between vancomycin and ototoxicity in infants and children. However, PK/PD studies have reported that an AUC_0–24_ > 400 was associated with higher bacteriological cure rates in CoNS bacteremia (38, 39). Further research is therefore needed to evaluate the clinical benefit of a loading dose strategy.

The final model aligns with the established understanding of vancomycin PK (40), retaining the primary covariates of renal function, body weight, and age-related maturation. Consistent with Colin et al. (12), the typical CL estimate of 0.108 L/h/kg was comparable to their reported value of 0.0758 L/h/kg, both falling within the estimated range for pediatric patients (0.0155–0.255 L/h/kg) in a systematic review by Aljutayli et al. (41). However, our volume of distribution estimate of 5.31 L/kg was at the upper end of the review range (0.32–5.89 L/kg) (41), likely attributable to the inclusion of critically ill ICU patients (28% of the cohort) known to experience fluid overload and capillary leak (42–45). Similar high estimates have also been reported in other pediatric cohorts, such as in children with malignant hematological diseases (13) and in infants (46). Although high shrinkage was observed for the IIV of V1, its impact on the model’s clinical application is likely minimal. Given that AUC_24_ is primarily determined by clearance rather than the central volume of distribution, the model remains a reliable tool for model-informed precision dosing (MIPD), where the goal is to achieve target therapeutic exposure. Additionally, concomitant nephrotoxic medications were not screened as covariates as their physiological impact is primarily captured by SCR levels, and including both would introduce significant collinearity. This aligns with a comprehensive review showing that such medications are rarely retained as significant covariates in pediatric vancomycin models once renal function is accurately modeled (41). While a study by Guilhaumou et al. (47) included cyclosporine as a covariate, their final model excluded SCR, suggesting the medication served primarily as a surrogate for renal impairment in their specific oncology cohort.

This study has several limitations. First, our model’s recommendations do not apply to all pediatric subgroups. Patients receiving ECMO, peritoneal dialysis, or RRT were excluded due to the significant pharmacokinetic alterations in these populations (48). Second, the study is also limited by its retrospective design, which may have led to variable sample timing and incomplete clinical documentation, such as the lack of measured creatinine clearance for evaluating augmented renal clearance. Third, potential heterogeneity may exist across the data sets due to differences in routine data collection and TDM practices at the external validation sites. Fourth, although the data used for model development were derived predominantly from a single pediatric center, this limitation is partly mitigated by the large sample size and the subsequent external validation using multicenter data. Finally, while the clinical uptake of MIPD software remains limited, our dosing tables provide a practical interim solution; to further support precision dosing, an online calculator based on this model is currently under development for the Kids Calc platform (https://www.kidscalc.org/).

### Conclusions

We successfully developed and externally validated a PopPK model for vancomycin in a general pediatric population. The resulting optimized dosing regimen achieved a high PTA of 73.0%. Prospective validation in clinical cohorts is needed in the future.

## Data Availability

Due to patient confidentiality, the clinical data collected in this study cannot be made publicly available.

## SUPPLEMENTAL MATERIAL

Fig. S1 aac.01840-25-s0001.tiff Distribution of sampling times after the previous dose in the development data set.

Supplemental material aac.01840-25-s0002.docx Tables S1 to S3; Supplemental figure caption.

ASM does not own the copyrights to Supplemental Material that may be linked to, or accessed through, an article. The authors have granted ASM a non-exclusive, world-wide license to publish the Supplemental Material files. Please contact the corresponding author directly for reuse.
