# Supplementary figures and images for: Model-informed precision dosing of vancomycin in children 3 months to 18 years of age using Australia-wide data
Source: Antimicrob Agents Chemother. 2026 May 12;70(6):e01840-25. doi: 10.1128/aac.01840-25 (PMC13231927; doi:10.1128/aac.01840-25)

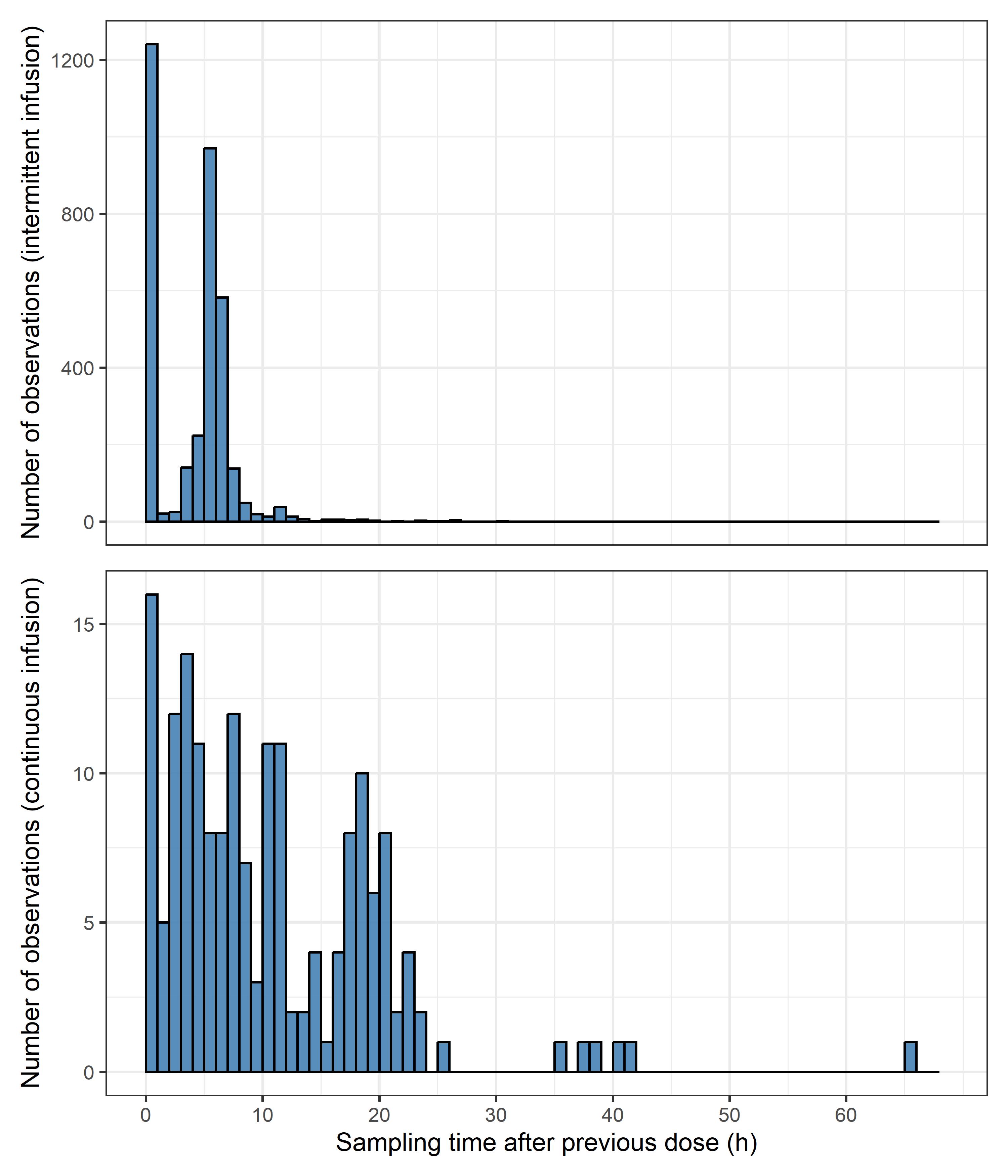

Supplement: Fig. S1 — Distribution of sampling times after the previous dose in the development data set. [file aac.01840-25-s0001.tiff]
