# Supplementary material for: Model-informed precision dosing of vancomycin in children 3 months to 18 years of age using Australia-wide data
Source: Antimicrob Agents Chemother. 2026 May 12;70(6):e01840-25. doi: 10.1128/aac.01840-25 (PMC13231927; doi:10.1128/aac.01840-25)
Supplement: Supplemental material — Tables S1 to S3; Supplemental figure caption. [file aac.01840-25-s0002.docx]

**Model-Informed Precision Dosing of Vancomycin in Children 3 Months to 18 Years of Age Using Australia-Wide Data**

Haiping Xu,^a^ Wenyu Yang,^a^ Tony Lai,^b,c,d^ Brendan McMullan,^e,f^ Daniel Yeoh,^g,h^ Amanda Wilkins,^i,j,k^ Zoy Goff,^g,l^ Xiao Zhu,^a,m^# Amanda Gwee,^i,j,k^#

^a^ Department of Clinical Pharmacy and Pharmacy Administration, School of Pharmaceutical Sciences, Fudan University, Shanghai, China

^b^Pharmacy Department, The Children's Hospital at Westmead, Sydney, NSW, Australia

^c^The University of Sydney Infectious Diseases Institute (Sydney ID), Sydney, NSW, Australia

^d^Sydney Pharmacy School, Faculty of Medicine and Health, The University of Sydney, Sydney, NSW, Australia

^e^Department of Infectious Diseases, Sydney Children’s Hospital, Randwick, NSW, Australia

^f^School of Clinical Medicine, The University of New South Wales, Sydney, NSW, Australia

^g^Department of Infectious Diseases, Perth Children's Hospital, Perth, WA, Australia

^h^Wesfarmers Centre for Vaccines and Infectious Diseases, Telethon Kids Institute, University of Western Australia, Perth, WA, Australia

^i^Department of General Medicine, Royal Children’s Hospital, Melbourne, VIC, Australia

^j^Department of Paediatrics, University of Melbourne, Parkville, VIC, Australia

^k^Antimicrobials Group, Murdoch Children’s Research Institute, Parkville, VIC, Australia

^l^Pharmacy Department, Perth Children's Hospital, Perth, WA, Australia

^m^National Key Laboratory of Advanced Drug Formulations for Overcoming Delivery Barriers, Shanghai, China

Running Head: Vancomycin MIPD in Australian Children

#Address correspondence to Xiao Zhu, [xiaozhu@fudan.edu.cn](mailto:xiaozhu@fudan.edu.cn) and Amanda Gwee, [Amanda.Gwee@rch.org.au](mailto:Amanda.Gwee@rch.org.au)

Haiping Xu and Wenyu Yang contributed equally to this work. Author order was determined alphabetically.

Table S1. Vancomycin dosing regimens, TDM practices, and analytical methods across the four participating centers

|  | RCH | PCH | SCH | CHW |
| --- | --- | --- | --- | --- |
| Starting dose of intermittent infusion | Intermittent (15mg/kg q6h) or continuous infusion | Intermittent (15mg/kg q6h) or continuous infusion | 15mg/kg/dose q6h (maximum: 750mg/dose) | 15mg/kg/dose q6h (maximum: 750mg/dose) |
| Lower limit of quantification (mg/L) | 5 | 1 | 4 | 5 |
| Types of TDM samples | Clinical troughs and random/peak samples | Clinical troughs and peak levels | Clinical troughs and random samples | Primarily clinical trough levels |
| Assay method and platform for vancomycin | VITROS Vancomycin Reagent (QuidelOrtho VITROS XT 7600) | Abbott Architect Reagent (Abbott Architect i2000) | KIMS Immunoassay (Roche Cobas Pure) | KIMS Immunoassay (Roche Cobas Pure) |
| Assay method and platform for serum creatinine | VITROS CREA Slide enzymatic assay (QuidelOrtho VITROS XT 7600) | Vitros Creatinine Reagent (Vitros 4600) | Enzymatic Method (Roche Cobas Pure) | Enzymatic Method (Roche Cobas Pure) |

RCH, Royal Children’s Hospital; PCH, Perth Children Hospital; SCH, Sydney Children’s Hospital; CHW, The Children's Hospital at Westmead; q6h, dosing every 6 hours; TDM, therapeutic drug monitoring.

Table S2. Development of final population pharmacokinetic model.

|  | **Structural model** | | **Base model** | | | | | | | | **Final model** |
| --- | --- | --- | --- | --- | --- | --- | --- | --- | --- | --- | --- |
|  | **1-comp** | **2-comp** | **1-comp + allometric scaling** | **2-comp + allometric scaling** | **2-comp + allometric scaling + maturation function (fixed)** | **2-comp + allometric scaling + updated maturation function (fixed)** | **2-comp + allometric scaling + maturation function (fixed) + SCR on CL** | **2-comp + allometric scaling + updated maturation function (fixed) + SCR on CL** | **2-comp + allometric scaling + maturation function (fixed) + SCR on CL + IOV on CL** | **2-comp + allometric scaling + maturation function (fixed) + SCR on CL + IOV on CL** | **2-comp + allometric scaling + maturation function (fixed) + SCR on CL + IOV on CL** |
| AIC | 15313.86 | 15303.27 | 14530.278 | 14407.53 | 14372.96 | 14387.58 | 12857.85 | 12905.478 | 12569.38 | 12682.537 | 12569.38 |
| OFV | 15303.86 | 15289.27 | 14520.278 | 14393.53 | 14358.96 | 14373.58 | 12841.85 | 12889.478 | 12545.38 | 12660.537 | 12545.38 |
| Minimization ^a^ | S, C | S, C | S, C | S, C | S, C | S, C | S, C | S, C | S, C | S, C | S. C |
| **Fixed effect parameters: Estimates (RSE)** | | | | | | | | | | | |
| CL (L/h) | 3.88 (3%) | 3.75 (3%) | 8.86 (2%) | 7.64 (3%) | 7.84 (4%) | 7.7 (3.5%) | 7.56 (3%) | 7.45 (3%) | 7.56 (3%) | 7.41 (3%) | 7.56 (3%) |
| V1 (L) | 41.3 (5%) | 40.9 (5%) | 109 (5%) | 104 (5%) | 103 (5%) | 104 (5%) | 84.4 (4%) | 85.6 (4%) | 85.6 (4%) | 74.9 (5%) | 85.6 (4%) |
| Q (L/h) | — | 0.134 (41%) | — | 1.42 (19%) | 1.47 (19%) | 1.43 (19.1%) | 1.3 (15%) | 1.27 (15%) | 1.3 (15%) | 1.29 (14%) | 1.3 (15%) |
| V2 (L) | — | 17.9 (15%) | — | 272 (28%) | 292 (29%) | 278 (28.6%) | 271 (31%) | 248 (30%) | 286 (31%) | 230 (32%) | 286 (31%) |
| SCR on CL | — | — | — | — | — | — | 0.0185 (2%) | 0.0183 (7%) | 0.0188 (7%) | 0.0182 (6%) | 0.0188 (7%) |
| WT on CL | — | — | 0.75 fixed | 0.75 fixed | 0.75 fixed | 0.75 fixed | 0.75 fixed | 0.75 fixed | 0.75 fixed | 0.75 fixed | 0.75 fixed |
| WT on V1 | — | — | 1 fixed | 1 fixed | 1 fixed | 1 fixed | 1 fixed | 1 fixed | 1 fixed | 1 fixed | 1 fixed |
| WT on V2 | — | — | 0.75 fixed | 0.75 fixed | 0.75 fixed | 0.75 fixed | 0.75 fixed | 0.75 fixed | 0.75 fixed | 0.75 fixed | 0.75 fixed |
| WT on Q | — | — | 1 fixed | 1 fixed | 1 fixed | 1 fixed | 1 fixed | 1 fixed | 1 fixed | 1 fixed | 1 fixed |
| Hill | — | — | — | — | 3.4 fixed(1) | 3.49 fixed(2) | 3.4 fixed(1) | 3.49 fixed(2) | 3.4 fixed(1) | 3.4 fixed(1) | 3.4 fixed(1) |
| TM_50_ (weeks) | — | — | — | — | 47.7 fixed(1) | 33.6 fixed(2) | 47.7 fixed(1) | 33.6 fixed(2) | 47.7 fixed(1) | 47.7 fixed(1) | 47.7 fixed(1) |
| PNAMAX | — | — | — | — | — | 0.588 fixed(2) | — | 0.588 fixed(2) | — | — | — |
| PNAT50 | — | — | — | — | — | 6.94 fixed(2) | — | 6.94 fixed(2) | — | — | — |
| **Inter-individual variability (CV%): Estimates (RSE) [Shrinkage]** | | | | | | | | | | | |
| CL | 71% (4%) [8%] | 74% (4%) [7%] | 39.5% (5%) [10%] | 47% (5%) [10%] | 45.8% (6%) [10%] | 46.4% (5.5%) [10%] | 27.1% (5%) [14%] | 28.4% (5%) [13%] | 22.1% (9%) [31%] | 20% (9%) [31%] | 22.1% (9%) [31%] |
| V1 | 84.4% (4%) [30%] | 85.1% (5%) [31%] | 50.4% (10%) [43%] | 49.7% (11%) [46%] | 49.2% (11%) [46%] | 49.4% (10.7%) [46%] | 30.4% (13%) [55%] | 31.4% (12%) [54%] | 32.9% (12%) [51%] | — | 32.9% (12%) [51%] |
| **Inter-occasion variability (CV%): Estimates (RSE) [Shrinkage]** | | | | | | | | | | | |
| CL | — | — | — | — | — | — | — | — | 18.7% (8.2%) [41%] | 17.9% (8%) [39%] | 18.7% (8.2%) [41%] |
| **Residual unexplained error: Estimates (RSE) [Shrinkage]** | | | | | | | | | | | |
| Prop.error | 0.293 (3%) [10%] | 0.292 (3%) [10%] | 0.297 (3%) [8%] | 0.291 (3%) [8%] | 0.291 (3%) [8%] | 0.291 (2.7%) [8%] | 0.254 (2.7%) [8%] | 0.254 (2%) [7%] | 0.233 (2%) [9%] | 0.245 (6%) [8%] | 0.233 (2%) [9%] |

a. S: minimization successful; C: covariance step successful.

CL, clearance; IOV, inter-occasion variability; CV%: coefficient of variability, expressed as $\sqrt{{omega}^{2}}\times100\%$; V1: central compartment volume of distribution; V2: peripheral compartment volume of distribution; WT: body weight; Q: intercompartment clearance from central compartment to first peripheral compartment; RSE: relative standard error; SCR: serum creatinine (μmol/L); Hill, defining the steepness of the non-linear relationship; TM50, the time to reach 50 % of adult clearance; PNA, postnatal age; PNAMAX, the fractional increase relative to the completion of the birth-associated component of maturation; PNAT50, the half time required to achieve 50% of this maturational change, and PNA is in days; —, Parameter not included in the respective model structure.

Table S3. Simulated vancomycin exposure (AUC_24–48_) following a loading dose of 25 mg/kg across different pediatric subgroups^a^

| **Age** | **Serum creatinine (μmol/L)** | **Maintenance dose (mg/kg)** |  | **PTA (%)** | | |
| --- | --- | --- | --- | --- | --- | --- |
|  |  |  |  | **AUC_24-48_^a^**  **(<400)** | **AUC_24-48_**  **(400–650)** | **AUC_24-48_**  **(>650)** |
| 3 months **–** 1 years | 10 **–** 30 | 22 |  | 31.0 | 67.0 | 2.0 |
| 1 **–** 4 years | 10 **–** 30 | 22 |  | 34.6 | 63.0 | 2.4 |
|  | 30 **–** 50 | 19 |  | 12.5 | 79.5 | 8.0 |
| 4 **–** 10 years | 20 **–** 40 | 21 |  | 19.4 | 74.4 | 6.2 |
|  | 40 **–** 60 | 16 |  | 14.3 | 80.1 | 5.6 |
| 10 **–** 16 years | 30 **–** 60 | 19 |  | 18.4 | 71.1 | 10.5 |
|  | 60 **–** 80 | 13 |  | 13.8 | 81.1 | 5.1 |
| 16 **–** 18 years | 50 **–** 80 | 15 |  | 20.7 | 72.9 | 6.4 |
|  | 80 **–** 110 | 10 |  | 19.1 | 77.6 | 3.3 |

1. The unit of AUC is mg·h/L. After each patient was administered a loading dose of 25 mg/kg, the optimized dose in Table 3 was used as the maintenance dose (dosing every 6 hours).

**Supplementary Figure Legends**

**Fig S1. Distribution of sampling times after the previous dose in the development data set.**

**REFERENCE**

1. Rhodin MM, Anderson BJ, Peters AM, Coulthard MG, Wilkins B, Cole M, Chatelut E, Grubb A, Veal GJ, Keir MJ, Holford NHG. 2009. Human renal function maturation: a quantitative description using weight and postmenstrual age. Pediatr Nephrol 24:67–76. https://doi.org/10.1007/s00467-008-0997-5
2. O'Hanlon CJ, Holford N, Sumpter A, Al-Sallami HS. Consistent methods for fat-free mass, creatinine clearance, and glomerular filtration rate to describe renal function from neonates to adults. 2023. CPT Pharmacometrics Syst Pharmacol 12(3):401–412. https://doi.org/10.1002/psp4.12924. Erratum in: CPT Pharmacometrics Syst Pharmacol. 2024 Jan;13(1):181–182.
